# The influence of traditional food consumption on Cameroonian immigrants’ perception of type 2 diabetes in Minnesota: an exploratory qualitative study

**DOI:** 10.3389/fpubh.2026.1767632

**Published:** 2026-03-16

**Authors:** Womma Habiba Hira, Maurine Ekun Nyok, Ngambouk Vitalis Pemunta, Brendabell Ebanga Njee, Mathias Fubah Alubafi, Nguyen Ngoc Bich Tram, Vidarah Nimar, Asahngwa Constantine Tanywe, Judith Fodjou, Tom Obara Bosire

**Affiliations:** 1 School of Public Health and Community Medicine, Goteborgs Universitet, Gothenburg, Sweden; 2 Department of Political Science, Univerzita Hradec Kralove, Hradec Kralove, Czechia; 3 Department of Sociology, Covenant University, Ota, Nigeria; 4 Cameroon Centre for Evidence-based Healthcare, Yaounde, Cameroon; 5 African Public Health Organization, St. Michael, MN, United States; 6 Human Sciences Research Council, Developmental, Capable, and Ethical State (DCES) Division, Pretoria, South Africa; 7 Centre for Gender and Africa Studies, University of the Free State, Bloemfontein, South Africa; 8 Department of Anthropology, Universite de Yaounde I, Yaoundé, Cameroon; 9 Department of Anthropology, Centre for Evidence-based Healthcare, Yaounde, Cameroon; 10 Department of Sociology, University of Glasgow, Glasgow, United Kingdom

**Keywords:** Cameroonian immigrants, culturally responsive health interventions, health behavior theories, traditional food consumption, type 2 diabetes risk perception

## Abstract

**Purpose:**

This study explored how traditional food consumption shapes perceptions of type 2 diabetes (T2D) risk among nondiabetic Cameroonian immigrants residing in Minnesota, USA. The focus was on participants’ dietary behaviors, lifestyle adaptations, and experiences with the U. S. healthcare system. Guided by an integrated theoretical framework—including the Health Belief Model (HBM), socioecological theory (SEM), risk/protective factors, and Social Cognitive Theory (SCT)—the study aimed to understand perceived influences rather than establish causal effects.

**Methods:**

Thirteen Cameroonian immigrants aged 25–50, residing in the U. S. for at least one year, participated in semi-structured, in-depth interviews. Discussions included dietary practices, access to traditional foods, recipe adaptations, exercise routines, and experiences with healthcare providers. Data were analyzed thematically using a rigorous coding process, with interpretations explicitly linked to the integrated theoretical frameworks to enhance credibility and theoretical coherence.

**Results:**

Participants retained strong cultural ties through traditional foods, but challenges such as limited availability, high cost, seasonal constraints, and competing work and family demands affected diet quality. Modifications to recipes and incorporation of locally available ingredients reflected behavioral flexibility and self-efficacy (SCT). Initial difficulties navigating the U. S. health insurance system highlighted structural barriers, while later understanding illustrated multi-level influences on health behaviors (SEM). Limited culturally appropriate healthcare services and gaps in knowledge about T2D risk underscored individual and community-level barriers, alongside protective factors such as family support, community networks, and motivation for healthier behaviors (HBM, risk/protective factors).

**Conclusion:**

Findings highlight how traditional food consumption correlates with perceptions of T2D risk and interacts with broader lifestyle, cultural, and healthcare contexts. While the study does not claim causality, qualitative data reveal pathways through which dietary practices inform health beliefs and decision-making within migrant contexts. These insights support the need for culturally responsive, multi-level interventions that integrate dietary counseling, lifestyle modification, and education. Clinicians and public health practitioners should adopt theoretically grounded, culturally sensitive strategies to enhance self-efficacy and support sustainable T2D prevention among Cameroonian immigrants while acknowledging their distinct social and environmental contexts.

## Introduction

Diabetes is a growing global health concern, affecting millions worldwide. In 2019, an estimated 463 million people (9.3%) had diabetes, with projections indicating an increase to 700 million (10.9%) by 2045 (1–3). More recent estimates indicate that over 830 million adults currently live with diabetes globally, with projections of 853 million by 2050 (4, 5). Type 2 diabetes (T2D) accounts for approximately 90% of all diabetes cases (3). In the United States, 34.2 million people (10.5%) have diabetes, including 7.3 million undiagnosed cases (6). Black or non-Hispanic African American adults experience a higher prevalence of diabetes (12.5%) compared with non-Hispanic Whites (7.5%), reflecting persistent health disparities (6, 7).

Immigrant populations are particularly vulnerable to diabetes and its complications. The African immigrant population in the United States increased substantially between 2008 and 2019 (8, 9). African immigrants, including Cameroonians residing in Minnesota, experience an increased risk of obesity and T2D following extended residence in the United States, largely due to lifestyle changes and dietary modification (10, 11). Long-term residence in the United States (over 10 years) has been associated with higher obesity rates among immigrant populations than among U. S.-born populations (10). Studies further indicate that African immigrants often adopt Western dietary patterns that are high in refined carbohydrates, partly due to limited access to affordable traditional foods, thereby exacerbating metabolic risk (12–15).

Given the growing size of the immigrant population and their elevated risk for diabetes, these trends have direct implications for national healthcare expenditure. In the absence of effective prevention strategies, the increasing prevalence of T2D among immigrants contributes substantially to the overall economic burden of diabetes in the United States (16, 17).

The rising diabetes burden carries substantial clinical and economic implications. In 2017, diabetes-related healthcare costs in the United States reached approximately $327 billion, including direct medical expenditures and reduced productivity (16). Preventive strategies emphasizing healthy eating and lifestyle modification are therefore essential (18). However, cultural context plays a central role in shaping dietary choices. Traditional foods are central to cultural identity and social life but may increase diabetes risk when consumed in excess, particularly carbohydrate-dense staples such as cassava-based fufu and white rice (14, 19). Beliefs, family roles, and social networks significantly influence perceptions of health and diet (20, 21). Recent evidence also highlights persistent knowledge gaps among immigrant communities regarding nutrition, carbohydrate content, and T2D risk (12, 22).

Minnesota was selected as the study site because it hosts a substantial and growing foreign-born population, with the seven-county Minneapolis–St. Paul metropolitan area serving as the primary hub for immigrants, including many from African countries such as Cameroon, making it a highly relevant context for examining dietary practices and health perceptions among Cameroonian immigrants (23, 24). Additionally, research on African immigrant health in Minnesota highlights the presence of diverse African immigrant groups and associated health risk factors, including cardiovascular conditions such as diabetes, underscoring the importance of studying chronic disease perceptions within this community (25, 26). Moreover, diabetes prevalence in Minnesota reflects national patterns, with approximately one in ten adults living with diagnosed diabetes, reinforcing the suitability of the state as a context for exploring health disparities and diabetes-related perceptions among minority and immigrant populations (27).

### Comparative context of African migrant health

The experiences of Cameroonian immigrants in Minnesota reflect broader patterns observed among sub-Saharan African migrants in high-income countries, including the United States (15, 26), Europe (13, 28), the United Kingdom, and Canada (28, 29). Similar to Nigerian and Ghanaian immigrants in the United States, participants in this study retained culturally significant, carbohydrate-rich traditional foods while also adopting elements of the host-country diet, reflecting a common dietary transition that contributes to increased metabolic risk (12, 13, 15, 30). Like sub-Saharan African communities in the United Kingdom, Cameroonian immigrants exhibited variability in knowledge and awareness of T2D, with some participants holding misconceptions about diet and disease while others actively seeking corrective information through community workshops or healthcare guidance (29, 31). These patterns highlight shared cultural influences, including the perception of traditional foods as inherently healthy and barriers to adopting dietary advice that conflicts with cultural norms.

However, important differences emerge across contexts. Cameroonian immigrants in Minnesota navigate a relatively smaller and more dispersed diaspora compared with larger, more established Ghanaian or Nigerian communities in European cities (13, 28, 32). This difference in community structure affects access to social support, communal food networks, and culturally tailored health information. Additionally, differences in length of residence and acculturation trajectories may influence dietary adherence: participants in Minnesota generally had shorter migration histories than European cohorts, shaping both retention of traditional diets and integration of host-country foods (26, 28, 32). Environmental factors, such as Minnesota’s climate and availability of traditional foods, further shape dietary adaptations in ways that differ from urban European settings examined in the RODAM studies (12, 13, 28, 30, 32–34).

Collectively, these findings position Cameroonian immigrants as both part of a broader African migrant health landscape—sharing patterns of dietary transition, cultural food norms, and challenges with health literacy—and distinct in terms of community structures, acculturation experiences, and environmental constraints. Recognizing these nuances is essential for designing culturally and contextually tailored interventions that address T2D risk while respecting the cultural identity and lived experiences of this population.

Many studies recommend that healthcare providers integrate culturally sensitive approaches that address dietary practices, family influences, and systemic barriers to improve diabetes prevention and management among immigrant populations (23–26). Strategies incorporating multi-level interventions—spanning individual, interpersonal, community, and policy levels—are increasingly recommended to mitigate T2D risk among high-risk immigrant groups (5, 17, 27).

This qualitative exploratory study examined the influence of traditional food consumption on perceptions of type 2 diabetes among Cameroonian immigrants in Minnesota, USA. The study contributes to the literature by focusing on an understudied African immigrant subgroup, applying an integrated theoretical framework (Health Belief Model, Social Ecological Model, and Social Cognitive Theory), and exploring how culturally embedded dietary practices intersect with perceptions of diabetes risk within the broader African migrant context. The research aimed to address existing gaps in understanding their views, barriers, and adaptation strategies related to traditional dietary practices, and to explore how these factors may contribute to risk factors for type 2 diabetes within this population.

### Objectives and research questions

The primary objective of this study was to explore how traditional food consumption shapes Cameroonian immigrants’ perceptions of their risk of developing T2D. Secondary objectives included examining dietary challenges in the U. S., identifying barriers to culturally appropriate healthcare, and understanding how lifestyle adaptations and social environments influence diabetes-related behaviors.

Guided by these aims—the study addressed the following research questions:

1. How do Cameroonian immigrants perceive the impact of traditional food consumption on their risk of developing T2D?
2. What specific challenges do Cameroonian immigrants face in maintaining traditional dietary practices in the United States, including availability, cost, and lifestyle constraints?
3. How do social networks, family responsibilities, and community environments shape dietary decisions and lifestyle changes relevant to T2D prevention?
4. What specific barriers do Cameroonian immigrants encounter when accessing culturally appropriate healthcare and navigating the U. S. health insurance system?

By addressing these questions, the study aimed to provide a nuanced understanding of the interplay between cultural dietary practices, social and environmental factors, and diabetes-related health behaviors. The following section describes the methods used to investigate these experiences and perceptions, guided by an integrated theoretical framework that considers both individual beliefs and broader socioecological influences.

## Methods

### Theoretical framework

This study draws on an integrated theoretical framework combining the Health Belief Model (HBM), the Socioecological Model (SEM), Social Cognitive Theory (SCT), and the risk and protective factors model. Together, these perspectives provide a multidimensional approach to understanding how individual beliefs, cultural orientations, social relationships, and structural environments influence dietary practices and T2D perceptions among Cameroonian immigrants in Minnesota.

### Health Belief Model (HBM)

The Health Belief Model (HBM), developed by Irwin M. Rosenstock in 1950, consists of several core concepts designed to predict individual behaviors related to illness prevention and management. These concepts include perceived susceptibility, perceived severity, perceived benefits and barriers to action, cues to action, and self-efficacy. According to HBM, individuals are more likely to adopt health-promoting behaviors if they recognize their susceptibility to a health condition, acknowledge the potential seriousness of the condition, believe that specific actions would reduce their risk or mitigate the condition’s impact, and determine that the benefits of those actions surpass any associated barriers or costs.

HBM explains how individual-level perceptions shape preventive behaviors, including perceived susceptibility, severity, benefits, and barriers. Studies demonstrate that immigrants’ perceived knowledge of diabetes and beliefs about diet strongly affect their engagement in prevention behaviors (2, 5, 23–27, 29). Although self-efficacy sometimes appears as a modifying factor within HBM, it is located conceptually and historically within SCT. In this study, HBM modifying factors include participants’ cultural health beliefs, prior experiences with diabetes, and their culturally grounded understanding of food and illness (27, 28).

#### Illustration

A Cameroonian immigrant who believes that “diabetes runs in the family” (perceived susceptibility) and fears complications such as blindness (perceived severity) may be more motivated to reduce sugar intake. However, lack of access to familiar foods (perceived barriers) may limit actual behavior change.

### Socioecological model (SEM)

The SEM situates individuals within overlapping social and environmental systems that collectively shape health behaviors (13). For Cameroonian immigrants, the migration process introduces new barriers to traditional dietary practice—such as limited access to traditional foods, higher food prices, and unfamiliar health insurance structures in the United States—that directly influence moderated daily choices and constraints (2, 25, 26). These structural pressures intersect with cultural norms, community expectations, and family dynamics, creating a complex environment in which dietary adaptation occurs.

For example, an individual may wish to prepare traditional dishes like eru or ndolé but discovers that key ingredients are either unavailable or significantly more expensive in mainstream U. S. grocery stores. This limited accessibility often necessitates dietary substitutions, which can shift eating patterns toward more accessible, calorie-dense American foods.

#### Risk and protective factors model

The risk and protective factors model identifies the conditions that heighten vulnerability or strengthen resilience across ecological levels (24, 25). Applying this model to the current study reveals several interrelated obstacles toward maintaining traditional food habits, including high food prices, limited availability of traditional food, socioeconomic constraints, and the lack of suitable healthcare services (2, 23, 25). At the same time, participants described multiple protective factors that help safeguard against these challenges, such as knowledge of traditional foods, strong family networks, active social ties, communal eating practices that reinforce healthy norms, and health literacy within the community (27–29). Collectively, these factors demonstrate how both structural barriers and cultural strengths shape Cameroonian immigrants’ dietary practices and health perceptions.

Importantly, these influences do not operate as a separate theoretical layer but intersect across individual, interpersonal, and environmental spheres. For example, community potlucks where participants share low-oil versions of traditional dishes promote healthier eating patterns while preserving cultural identity.

### Social cognitive theory (SCT)

SCT offers a valuable framework for understanding how health behaviors are learned, practiced, and reinforced across social contexts (7, 8). A core premise of SCT is that individuals do not make decisions alone; instead, their actions are shaped by ongoing interactions between personal beliefs, social environments, and the behaviors they observe within their communities (7, 9). Two SCT constructs are particularly relevant to this study. The first is observational learning, through which individuals acquire new recipes, experiment with healthier ingredient substitutions, or learn strategies for navigating the U. S. healthcare system by watching how others in their community manage similar situations (2, 25). The second is self-efficacy, defined as one’s confidence in the ability to adopt and sustain healthy behaviors despite barriers. Previous studies have found evidence that links higher self-efficacy to more effective diabetes self-management and stronger engagement in preventive practices (23, 29, 35).

SCT also introduces the concept of reciprocal determinism, which highlights the dynamic, bidirectional relationship between individuals, their environments, and their behaviors (7, 8). This interplay can be seen when a participant observes a friend successfully replacing palm oil with olive oil—an instance of observational learning that may motivate the individual to try the same substitution. As their confidence grows and the behavior becomes routine, it can begin to influence the household’s cooking norms, illustrating how behavior change unfolds through continuous feedback within social systems.

### Integrated interpretation

Integrating these four theories produces a broader and more culturally attuned framework than any single model could achieve. The HBM illuminates how individuals interpret their own diabetes risk and how these perceptions shape dietary decisions (5, 23, 35). The SEM complements this by situating individual beliefs within the wider cultural, environmental, and structural contexts that influence daily behavior (2, 25, 26). SCT adds insight into how health behaviors are learned, reinforced, and sustained through social interactions and observation (7–9). The risk and protective factors model further enhances the framework by identifying the conditions that either intensify vulnerability or bolster resilience across ecological levels (24, 25). Taken together, these perspectives offer a comprehensive, culturally responsive, and ecologically grounded approach for interpreting Cameroonian immigrants’ dietary practices and their perceptions of T2D in the U. S. context.

**Figure 1:**
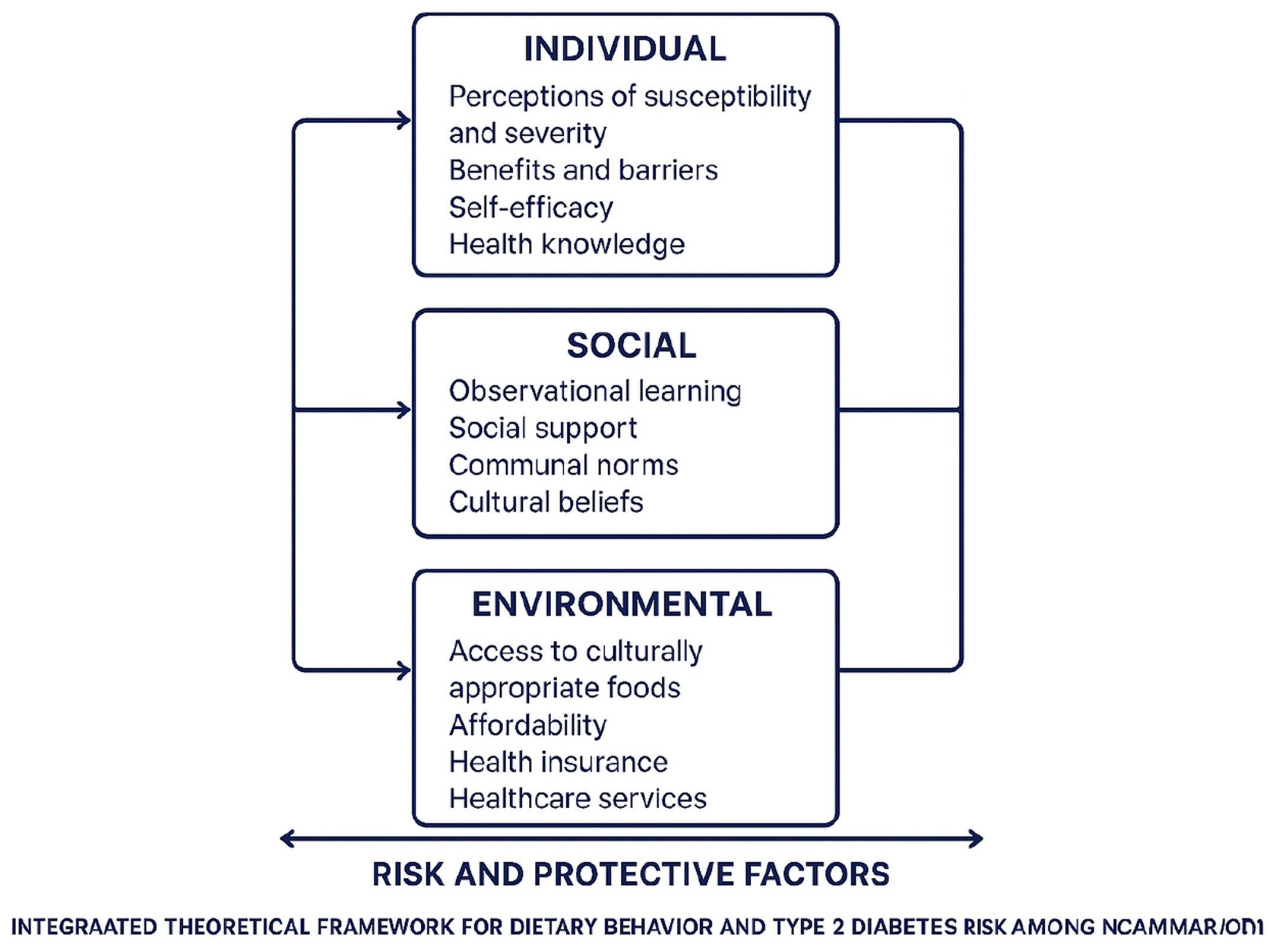
Integrated theoretical framework.

Figure 1 illustrates how individual, social, and environmental levels (SEM) interact with constructs from the HBM, SCT, and the risk and protective factors model to shape dietary behaviors and perceptions of T2D risk among Cameroonian immigrants. Arrows represent multidirectional influences consistent with reciprocal determinism (7, 8), and risk/protective factors (24, 25) operate across levels rather than as a separate hierarchical layer.

### Research design

This qualitative study employed an exploratory research design, appropriate for exploring perceptions, beliefs, lived experiences, and health behaviors of individuals. This research design is flexible and capable of addressing complex issues, making it suitable for examining health beliefs, dietary practices, and migration-related adaptations of Cameroonian immigrants in Minnesota. The study was guided by the integrated theoretical framework combining HBM, SEM, the risk and protective factors model, and SCT.

Semi-structured, face-to-face interviews were used to allow flexibility in exploring participants’ perceptions, beliefs, and lived experiences, while fostering personal dialogue and narratives. This approach aligns with qualitative principles by capturing rich verbal accounts and context-specific cultural knowledge, enabling examination of how individual beliefs, social networks, and environmental contexts influence dietary behaviors and T2D risk.

An interview guide (Appendix 1) provided a structured framework to ensure consistency across interviews while allowing contextual adaptations during data collection. This flexible structure ensured both comparability and cultural responsiveness, permitting participants’ real-life experiences to guide the depth and direction of discussion.

### Study sample

A purposive sampling strategy was used to recruit 13 non-diabetic Cameroonian immigrants aged 25–50 years who had resided in Minnesota for a minimum of two years. Ethical approval was obtained from Walden University Institutional Review Board (IRB Approval 01–04–19-049877), and written informed consent was obtained from all participants prior to interviews. Participants were informed of their rights to withdraw at any time without consequence.

Consistent with qualitative research standards, a sample size of at least 12 participants is widely accepted as adequate for achieving data saturation, where no new themes emerge after repeated interviews. In this study, saturation was assessed iteratively during data collection by reviewing emerging codes and themes after each interview. Saturation was reached after the 12th interview, with the 13th participant included to enhance thematic diversity and analytical rigor, particularly given the heterogeneity of the Cameroonian immigrant population with respect to socioeconomic status, acculturation, and length of stay (30).

The slightly larger sample size allowed exploration of variations in cultural beliefs, social networks, and lifestyle practices, supporting the examination of differences in perceived susceptibility, perceived severity, perceived benefits and barriers, and behavioral adaptation as informed by the HBM and socioecological perspectives.

### Pretesting of the interview guide

The interview guide was pretested with two participants to assess clarity, relevance, and construct validity (31, 32). The pretest confirmed the guide’s effectiveness in capturing key constructs derived from HBM, SEM, SCT, and the risk/protective factors model. Feedback from the pretest led to minor refinements in question order and phrasing for improved flow. Because the pretest data were relevant, theoretically aligned, and collected using the same interview protocol, they were incorporated into the final dataset (34).

### Data collection and procedure

Interviews were conducted in 2023 at participants’ homes in English. Topics included dietary practices, physical activity, challenges to maintaining healthy lifestyles, and experiences related to preserving traditional diets in the U. S. Contextual factors such as perceived benefits and barriers, cultural beliefs, family support, community resources, and knowledge of T2D were explored. Open-ended questions enabled participants to describe how personal beliefs, observational learning, and social networks influenced dietary behaviors.

### Data analysis

Data were analyzed thematically using the six-phase approach described by Braun and Clarke (41), which provides a systematic and flexible approach for identifying, organizing, and interpreting patterns of meaning across qualitative findings. The analysis was iterative and interpretive, and was informed by the integrated theoretical framework combining HBM, SEM, SCT, and the risk and protective factors model.

#### Phase 1: familiarization with the data

All interviews were transcribed verbatim and repeatedly read to ensure deep immersion in the data. Audio recordings were re-listened to capture nuances in tone, emphasis, and cultural expressions. Initial notes were made on participants’ perceptions of T2DM, cultural beliefs, migration-related dietary adaptations, and contextual barriers and facilitators influencing health behaviors.

#### Phase 2: generating initial codes

Transcripts were coded using both inductive codes emerging from participants’ narratives and deductive codes guided by the theoretical framework (e.g., perceived susceptibility, barriers, observational learning, environmental constraints, protective resources). Coding was conducted systematically across all transcripts to ensure comprehensive coverage. Reliability and clarity were ensured by regular peer discussion of the research group to avoid any discrepancy among coders.

#### Phase 3: searching for themes

Codes were clustered into broader candidate themes reflecting patterned meanings across participants. Candidate themes included: (a) migration and dietary adaptation, (b) beliefs and perceptions of T2DM risk, (c) structural and environmental constraints, and (d) social networks and cultural identity as protective resources. Relationships between codes and themes were visually mapped to explore intersections of individual beliefs, social influences, and environmental contexts.

#### Phase 4: reviewing themes

Themes were reviewed for internal coherence and analytical distinctiveness. The dataset was revisited to ensure themes accurately represented participants’ accounts and were sufficiently supported by data extracts. Some themes were refined, collapsed, or expanded to improve conceptual clarity, ensuring alignment with the theoretical framework.

#### Phase 5: defining and naming themes

Final themes and subthemes were clearly defined, with each theme capturing a meaningful aspect of participants’ perceptions, experiences, and adaptation strategies. Subthemes were created where necessary to reflect nuanced patterns such as *substitution strategies*, *cultural food preservation*, and *exposure to fast foods*.

#### Phase 6: producing the report

Illustrative quotations were selected to support themes, and findings were interpreted in relation to the integrated theoretical framework. This process demonstrated how individual beliefs (HBM), social and environmental contexts (SEM), behavioral learning (SCT), and cross-level risk/protective factors intersect to shape dietary behaviors and perceptions of T2DM risk among Cameroonian immigrants in Minnesota.

### Ethical considerations

Prior to data collection, participants were approached in person and provided with a thorough explanation of the study’s objectives, procedures, and expectations. Written informed consent was obtained from all participants. The consent form clearly outlined the study purpose, data collection methods, potential risks and benefits, and the measures implemented to ensure confidentiality and secure data management.

The study was conducted in full compliance with the ethical principles of the Declaration of Helsinki (40) and the Belmont Report. Ethical approval was granted by the Walden University Institutional Review Board (IRB Reference: 01-04-19-049877).

To protect participant anonymity, pseudonyms were assigned, with identifiers beginning with “J” for male participants and “M” for female participants. All recordings, transcripts, and study materials were securely stored on an external, password-protected drive accessible only to authorized research team members. Participants were informed that their participation was voluntary and that they could withdraw at any time without penalty.

The interdisciplinary composition of the research team—including expertise in sociology, psychology, healthcare administration, global health, arts history, and business management—enhanced ethical oversight and promoted reflexivity throughout the study. Regular reflexive discussions were conducted during data collection and analysis to ensure culturally sensitive engagement, minimize disciplinary biases, and accurately represent participants’ perspectives. This approach aligns with the study’s theoretical framework, particularly the Socioecological Model and Social Cognitive Theory, emphasizing respect for the contextual, relational, and cultural dimensions of participants’ lived experiences.

With these ethical safeguards in place, participants’ perspectives were carefully documented, providing a foundation for the study findings on dietary practices and type 2 diabetes risk among Cameroonian immigrants.

## Results

This section presents the themes that emerged from the qualitative exploration of how traditional food consumption shapes nondiabetic Cameroonian immigrants’ perceptions of T2D in Minnesota. Guided by a phonetic iterative coding approach, the analysis moved iteratively between the data, relevant theory, and emerging interpretations to arrive at six consolidated themes. Several initially overlapping categories were merged to enhance conceptual clarity and to better reflect the interconnected nature of participants’ lived experiences.

The first theme examines participants’ dietary and physical activity behaviors, highlighting how embodied habits, cultural food practices, and daily routines influence their understanding of metabolic health. The second theme explores lifestyle adaptations, showing how migration and exposure to new social environments shape dietary patterns, food preparation, and physical activity. A third theme addresses participants’ knowledge and awareness of T2D, including how they interpret risk, symptoms, prevention, and the relevance of medical guidance.

The fourth theme focuses on practical challenges navigating food access and healthcare systems in Minnesota, including barriers to obtaining traditional foods and accessing culturally relevant health information. The fifth theme explores cultural perceptions and motivations, illustrating how beliefs, values, and identity influence health behaviors and how these perceptions sometimes diverge or conflict among community members. Finally, the sixth theme captures community and social influences, demonstrating how family, peers, diaspora associations, and broader community norms shape dietary decisions and perceptions of diabetes risk.

Each theme is presented with illustrative participant quotations to foreground the voices and experiences of Cameroonian immigrants. Interpretive commentary accompanies the quotes, highlighting perceived risk, protective behaviors, and culturally embedded beliefs. Contradictory perspectives are explicitly included to reflect the diversity of views and experiences within the community.

### Theme 1: dietary and physical activity behaviors

#### Subtheme 1a – traditional food consumption

Participants expressed a strong preference for traditional foods, describing them not only as familiar and comforting but also as inherently nutritious and closely tied to cultural identity. These foods served as symbols of heritage, family continuity, and communal belonging. Many participants emphasized that preparing and eating traditional meals helped them maintain a sense of connection to their homeland despite living abroad.

- Mary: “I like to eat legumes and nuts, like fufu with njamanjama.”
- Martha: “I eat vegetables like bitter leaves, which we mostly buy from Cameroon.”
- Jacob: “We eat vegetables from Africa, such as eru and pumpkin. Sometimes we get dried bitter leaves from Cameroon.”
- Contradictory vignette: Samuel: “I try to eat smaller portions of fufu now because I’ve heard it can increase blood sugar.”

Interpretive note: While most participants maintain traditional foods as a source of comfort and identity, others actively modify consumption due to health concerns, reflecting diverse approaches to balancing cultural practices and metabolic risk.

#### Subtheme 1b – physical activity

Participants engaged in a range of exercises, combining traditional movement practices with Western fitness routines. Levels of activity varied widely across participants, influenced by motivation, time availability, and environmental constraints.

- Mary: “I try to dance and walk at least three times a week.”
- Maggie: “I do treadmill, yoga, and dance three to four days a week.”
- Contradictory vignette: *“I barely have time to walk with my work schedule.”*

Interpretive note: These accounts show that while some participants integrate regular exercise into their routines, others face structural or occupational barriers that limit activity, highlighting the heterogeneity of physical activity strategies within the community.

### Theme 2: lifestyle adaptations

#### Subtheme 2a – dietary adaptations

Participants showed resourcefulness in sourcing and preserving traditional ingredients, navigating both environmental and financial constraints.

- Megan: “I eat bitter leaf soup and huckleberry during summertime and process to keep in the fridge for winter.”
- Jacob: “We have been able to defeat that challenge because we have resolved to buy some local things.”
- Contradictory vignette: Jacob: “I used to eat fufu and njamanjama every day, but I’ve started reducing the portions because it’s expensive and I worry it might raise my blood sugar. Sometimes I replace it with more vegetables or local grains.”

Interpretive note: This contrast demonstrates that adaptation strategies vary across participants. Some preserve traditional diets fully, while others strategically adjust portion sizes or substitute ingredients to balance cultural fidelity with health considerations.

#### Subtheme 2b – physical and environmental adjustments

Participants’ physical activity patterns were influenced by Minnesota’s climate, urban infrastructure, and neighborhood walkability. Seasonal weather and access to recreational spaces affected when and how they exercised.

- Megan: “In winter, it’s hard to get outside for exercise due to the cold and my night shifts.”
- Contradictory vignette: Mary: “Winter is really cold here, so I cannot run outside like I used to in Cameroon, but I joined a local community dance group and follow online workout videos at home to stay active.”

Interpretive note: While environmental barriers limit activity for some, others creatively adapt by finding indoor or community-based alternatives, highlighting variability in physical activity practices.

### Theme 3: knowledge and awareness of T2D

#### Subtheme 3a – knowledge

Participants’ understanding of T2D ranged from accurate biomedical knowledge to partial or culturally constructed misconceptions. Some linked T2D risk to diet, genetics, and lifestyle, while others focused on a single dietary factor.

- Magdalene: “Once you have type 2 diabetes, your pancreas does not produce or regulate enough insulin… you must rely on medications or insulin.”
- Jacob: “I do not know about the different levels, type 1 and 2… sometimes people need sugar in their blood.”
- Contradictory vignette: John: “I used to think fufu was safe in any amount, but after attending a nutrition workshop at our community center, I now limit portion sizes and balance it with more vegetables. I realized it could affect my blood sugar if I eat too much.”
- Contradictory vignette: Mary: “At first, I did not know that sugary drinks could increase diabetes risk. After talking to a nurse at a health fair, I started checking labels and reducing sodas.”

Interpretive note: These accounts demonstrate that while some participants hold misconceptions, others actively seek corrective information, reflecting variability in health literacy, exposure to educational resources, and proactive behavior change.

#### Subtheme 3b – awareness

Participants’ awareness of T2D varied in scope and depth. Some recognized risk factors affecting the broader community, while others had limited understanding or perceived the condition as stigmatized.

- Mary: “When I see a lot of people from my community indulging in drinking, I feel bad about it.”
- Maggie: “People do not like to talk about diabetics.”

Interpretive note: Silence or stigma around diabetes within the community may hinder information dissemination, complicating preventive efforts.

### Theme 4: experiences navigating food access and healthcare

#### Subtheme 4a – food access challenges

Participants reported difficulties in obtaining traditional foods due to cost, distance, and time constraints.

- Mary: “Traditional food is expensive, and sometimes we have to drive far to buy what we cannot find locally.”

#### Subtheme 4b – healthcare experiences

Participants described variable experiences with healthcare providers. Many received generic advice unfamiliar with traditional diets.

- Maggie: “They never included traditional foods; some lists were unfamiliar.”
- John: “I was told to cut down on rice, which I like to eat.”
- Contradictory vignette: Marie: “My doctor asked me about the foods I usually cook from home. She helped me adjust recipes by reducing oil and salt but still keeping my traditional vegetables and stews. It felt helpful because I did not have to give up the foods I love.”

Interpretive note: These examples highlight disparities in provider cultural competency and the value of tailored dietary guidance in supporting culturally congruent health behavior changes.

### Theme 5: cultural perceptions and motivations

#### Subtheme 5a – cultural significance of food

Participants emphasized that traditional diets are integral to cultural identity, family heritage, and perceived nutritional value.

- Monica: “We eat African food not because they are different but because we prepare them in our own way. We believe in organic and healthy food.”

#### Subtheme 5b – motivation for healthy Behaviors

Participants’ motivation to adopt healthier behaviors was influenced by individual goals, family expectations, and community norms.

- Maggie: “I think professional advice can help maintain healthy eating habits.”
- Miranda: “For me family education such as balancing njamanjama with less fufu corn is important.”
- Contradictory vignette: Joe: “I want to eat less fufu and oily meats to manage my blood sugar, but my family expects me to join them for big traditional meals. Sometimes I feel guilty if I do not eat as much as they do, so it’s hard to stick to healthier portions.”

Interpretive note: This illustrates the tension between personal health goals and cultural/family expectations, emphasizing the importance of family dynamics in dietary decision-making.

### Theme 6: community and social influences

Social networks played a key role in shaping dietary choices, reinforcing healthy behaviors, and facilitating knowledge exchange.

- Miranda: “My relatives taught me to reduce carbohydrate-heavy meals.”
- Jacob: “I think we have to reduce oil and meat consumption at home.”
- Contradictory vignette: Jacob: “My relatives keep telling me to reduce fufu and fatty foods, but I feel that these dishes are part of who I am. Sometimes I follow their advice, but other times I eat the traditional way because it feels right to me.”

Interpretive note: Community advice can act as a protective factor; however, individual beliefs and cultural preferences may lead participants to selectively follow or resist recommendations, illustrating variability in how social networks influence dietary behaviors.

Collectively, these findings highlight the complex interplay between cultural identity, dietary practices, environmental constraints, knowledge, and social influences in shaping perceptions of T2D risk among Cameroonian immigrants in Minnesota. Participants demonstrated a range of adaptive strategies, from preserving traditional foods to modifying portion sizes or adopting alternative physical activities, reflecting both resilience and variability in health behaviors. Contradictory perspectives—such as selective adherence to community advice, negotiation between family expectations and personal goals, and proactive engagement with corrective health information—underscore the heterogeneity of experiences within this population. These insights provide a nuanced foundation for the Discussion, where we interpret these patterns in relation to the theoretical frameworks (HBM, SEM, SCT) and existing literature, and for the Recommendations, which will address culturally tailored interventions that are sensitive to these lived experiences.

## Discussion of findings

### Dietary and physical activity behaviors

Participants consistently emphasized adherence to traditional foods while engaging in diversified physical activities such as walking, jogging, dance, and yoga. These behaviors reflect HBM constructs, including perceived benefits of traditional food and exercise. Findings reflected perceived barriers related to access, cost, and environmental constraints. SEM situates these behaviors within individual, interpersonal, community, and policy-level influences, such as family support, availability of traditional food, and seasonal factors.

These findings align with evidence of nutrition transition among West African migrants documented by Agyemang et al. (31, 33), who reported higher obesity and T2D prevalence among Ghanaian migrants in Europe compared to rural populations, highlighting how migration reshapes dietary risk profiles despite continued reliance on traditional foods. SCT mechanisms were evident in participants’ observational learning from family and peers and self-efficacy in maintaining culturally congruent health practices. Consistent with dietary acculturation research by Osei-Kwasi et al. (30), participants retained traditional staples while selectively incorporating host-country foods, reflecting a hybrid dietary pattern rather than wholesale dietary replacement. Protective factors including family encouragement, access to transnational foods, and cultural food knowledge reinforced these adaptive behaviors, while environmental constraints—particularly Minnesota’s cold climate and limited availability of some traditional foods—introduced context-specific limitations less emphasized in European studies.

### Lifestyle adaptations

Participants reported diverse strategies to adapt their diets and exercise routines in response to structural and environmental constraints. Seasonal planning, such as purchasing fresh products in summer and freezing for winter use, demonstrates behavioral flexibility and the ability to modify practices without compromising cultural identity.

From the HBM perspective, participants perceived these adaptations as necessary to overcome barriers while maintaining health. SEM highlights structural and social factors, including transportation challenges, neighborhood infrastructure, and familial support, which shaped participants’ capacity to implement changes. These adaptive practices echo broader patterns of migrant resilience described in African migrant literature, where dietary continuity is preserved through creative substitutions and seasonal planning despite constrained food environments (30, 31, 33). SCT constructs such as self-efficacy and modeling reinforced the successful implementation of these strategies, particularly within households and informal community networks.

### Knowledge and awareness of T2D

Knowledge about T2D varied widely among participants, ranging from comprehensive biomedical understanding to limited or fragmented awareness. HBM constructs—perceived susceptibility, perceived severity, and cues to action—explained how differences in knowledge influenced preventive behaviors. Participants with greater understanding reported more proactive engagement in dietary management and physical activity, while those with limited awareness expressed vulnerability to risk.

SEM situates knowledge and awareness within community norms and social networks, showing how education and family interactions serve as protective factors against unhealthy dietary transitions. This variability closely parallels qualitative findings by de-Graft Aikins et al. (35), who documented the coexistence of cultural explanations and biomedical knowledge of diabetes among sub-Saharan African migrant communities. SCT highlights observational learning, as participants learned about T2D through family, social networks, and healthcare providers. These findings reinforce that diabetes knowledge among African migrants is plural, negotiated, and unevenly translated into preventive action, rather than absent.

### Experiences navigating food access and healthcare

Participants described structural and practical challenges in maintaining traditional diets and accessing culturally competent healthcare. HBM constructs framed these experiences as perceived barriers to preventive behaviors. SEM situates these experiences within organizational and policy-level contexts, including healthcare accessibility, provider cultural competence, and socioeconomic constraints.

Risk factors such as language barriers, cultural discordance with providers, and limited food availability were balanced by protective factors including family and community support. SCT elucidates how exposure to culturally relevant role models influenced participants’ self-efficacy and behavioral choices. Comparable barriers to healthcare access have also been documented among Ghanaian migrants in Europe, where Marzà-Florensa et al. (33) highlight the role of community networks in mitigating challenges and shaping responses to multimorbidity and chronic disease management. Similarly, de-Graft Aikins et al. (35) show that social support not only facilitates access to culturally relevant health information but also influences how biomedical and culturally informed understandings of disease are negotiated within migrant communities. Together, these studies underscore that social networks function both as compensatory resources in the face of structural barriers and as critical conduits for health knowledge and preventive behaviors among African migrants.

### Cultural perceptions and motivations

Participants’ perceptions of traditional foods were closely tied to cultural identity and health beliefs. Traditional meals were seen as both nutritious and protective, while participants simultaneously recognized potential risks, such as high carbohydrate content. HBM constructs such as perceived benefits and cues to action explain participants’ motivation to maintain traditional diets alongside lifestyle modifications.

SEM highlights the influence of interpersonal networks and community norms, while SCT demonstrates the role of modeling and self-efficacy in motivating sustained engagement in health-promoting behaviors. These findings resonate with Osei-Kwasi et al.’s (30) observation that food practices among Ghanaian migrants are deeply embedded in identity maintenance, with dietary change negotiated rather than imposed. Protective factors, including family encouragement and cultural knowledge, enhanced motivation, whereas social expectations and conflicting health messages moderated preventive action.

### Community and social influences

Social networks and community engagement emerged as critical facilitators of preventive behaviors. Participants shared knowledge, modeled healthy behaviors, and encouraged dietary adherence within families and peer groups. HBM cues to action were reinforced through these social interactions, while SEM situates behaviors within broader community and cultural contexts.

Protective factors, such as family support and peer encouragement, strengthened self-efficacy and motivation (SCT), whereas risk factors like social isolation or stigma impeded engagement. Consistent with Marzà-Florensa et al. (33), community support functioned as a central mechanism through which chronic disease risk is collectively interpreted and managed among African migrants. These findings underscore that preventive behaviors are socially embedded rather than individually determined.

### Synthesis across themes

Consolidating overlapping themes highlights a dynamic interplay of individual beliefs, social networks, and environmental contexts. Across all themes, the experiences of Cameroonian immigrants reflect broader African migrant health trajectories, including nutrition transition, plural health knowledge, and reliance on community support, as documented among Ghanaian and other West African migrant populations (31, 33). At the same time, the Minnesota context introduces distinctive environmental and structural constraints—such as climate, food availability, and smaller diaspora communities—that shape how these broader patterns are locally enacted.

Taken together, these findings position Cameroonian immigrants as both part of a wider African migrant health landscape—sharing dietary transitions, cultural food norms, and challenges with health literacy—and as distinct in terms of local environmental constraints, community structure, and acculturation pathways. Recognizing these nuances is critical for designing culturally and contextually tailored interventions that address T2D risk while respecting cultural identity and lived experiences (Table 1).

**Table 1:** Mapping themes to integrated theoretical framework.

Theme	HBM	Socioecological model (SEM)	Risk/protective factors	Social cognitive theory (SCT)
Dietary & physical activity behaviors	Perceived benefits of traditional foods; perceived barriers to healthy eating and exercise	Individual, interpersonal, community, and policy-level influences on diet and activity	Protective: family support, access to traditional foods; Risk: environmental constraints, cold climate	Observational learning from family/community; reciprocal determinism shaping behavior; self-efficacy in sustaining behaviors
Lifestyle adaptations	Perceived ability to modify behaviors; cues to action	Physical environment, policy, and social support influencing adoption	Protective: social support, knowledge of healthy substitutions; Risk: financial and logistical barriers	Modeling adaptive behaviors; self-efficacy in implementing lifestyle changes
Knowledge & awareness	Perceived susceptibility and severity; cues to action	Community norms, cultural beliefs, and social networks	Protective: access to accurate information, peer education; Risk: misinformation, cultural myths, stigma	Observational learning through social networks; modeling health behaviors
Experiences navigating food & healthcare	Perceived barriers to preventive care	Organizational and interpersonal influences on healthcare access	Protective: family/community support; Risk: language/cultural barriers, limited provider cultural competence	Self-efficacy shaped by exposure to culturally competent role models; observational learning
Cultural perceptions & motivation	Perceived benefits and cues to action	Interpersonal networks, community norms, and cultural identity	Protective: cultural knowledge, family encouragement; Risk: social pressure or stigma	Self-efficacy and modeling reinforce motivation; goal-setting for health behaviors
Community & social influences	Cues to action; perceived benefits	Influence of family, peers, and community on behavior	Protective: peer/family encouragement, knowledge-sharing; Risk: social isolation, cultural stigma	Observational learning, modeling, social reinforcement of adaptive behaviors

**Figure 2:**
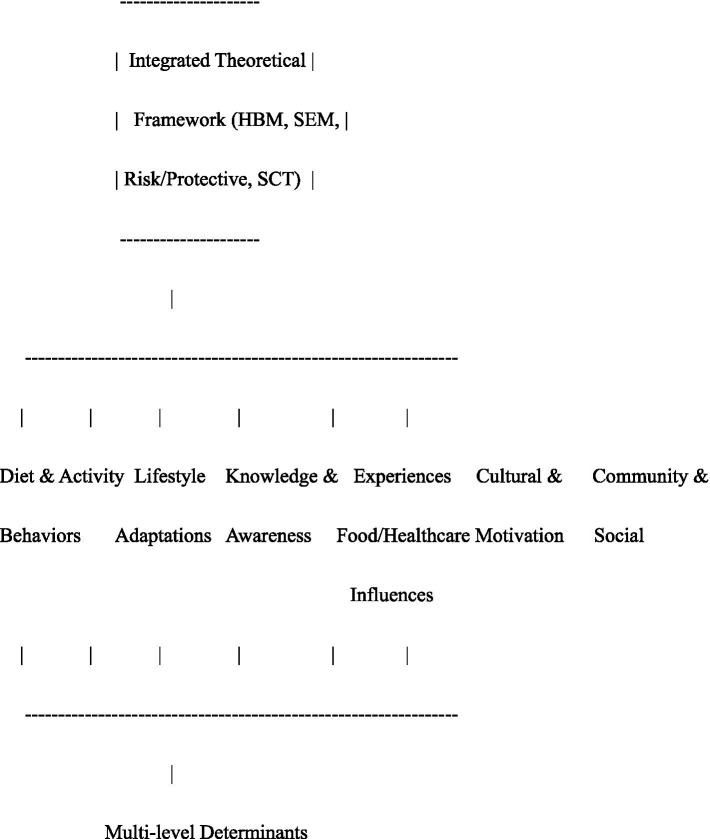
Visual concept of themes within integrated framework.

The Figure 2 is structured to show how the study’s analytical framework operates across different levels. At the top is the integrated theoretical framework that guides the overall interpretation of the findings. The middle section presents the consolidated themes and subthemes that emerged from the results. At the bottom, the figure highlights the cross-cutting influence of multi-level determinants, illustrating how factors at various ecological levels intersect to shape participants’ experiences. The interpretation of these relationships—and their connections to HBM, SEM, SCT, and the risk/protective factors model—is addressed below.

Explanation:

- Top box: Integrated theoretical framework guiding analysis.
- Middle row: Six consolidated themes emerging from the data.
- Bottom box: Cross-cutting influences (individual, social, and environmental) highlighting multidimensional determinants of T2D perceptions and behaviors.
- Arrows indicate reciprocal interactions consistent with SCT, while all themes acknowledge risk and protective factors operating across levels.

### Limitations

This study has several limitations that should be considered when interpreting the findings. First, the qualitative and exploratory design, while well suited for capturing lived experiences, cultural meanings, and nuanced perceptions of type 2 diabetes (T2D), limits the generalizability of the findings beyond the study population. The experiences described reflect those of a specific group of Cameroonian immigrants residing in Minnesota and may not fully represent Cameroonian immigrants in other U. S. states or African immigrant populations more broadly.

Second, the study relied on self-reported data, which may be subject to recall bias or social desirability bias. Participants may have underreported unhealthy dietary practices or overemphasized behaviors perceived as socially acceptable, particularly when discussing sensitive topics such as food choices, physical activity, or diabetes risk. However, the use of in-depth interviews and iterative probing helped to contextualize responses and identify internal contradictions, thereby strengthening the credibility of the findings.

Third, the sample size, while appropriate for qualitative inquiry, was relatively small and may not capture the full heterogeneity of experiences within the Cameroonian diaspora. Differences related to region of origin within Cameroon, socioeconomic status, immigration pathway, length of residence in the United States, and legal or employment status may shape dietary practices and health perceptions in ways not fully explored in this study.

Fourth, this study focused on nondiabetic participants’ perceptions of T2D risk and prevention. While this focus provides valuable insight into preventive beliefs and behaviors, it excludes the perspectives of individuals living with diagnosed T2D, whose experiences with disease management, healthcare systems, and dietary modification may differ substantially. Future research incorporating both preventive and clinical perspectives could offer a more comprehensive understanding of diabetes across the disease continuum.

Fifth, the cross-sectional nature of the study limits the ability to assess changes in dietary practices, health beliefs, or risk perceptions over time. Migration, acculturation, and exposure to healthcare systems are dynamic processes, and longitudinal research would be better positioned to capture how perceptions of traditional foods and T2D risk evolve with increased duration of residence in the United States.

Finally, while the study employed an integrated theoretical framework (Health Belief Model, Social Ecological Model, and Social Cognitive Theory) to guide analysis, theoretical integration may have shaped the interpretation of findings in ways that foreground certain constructs, such as perceived risk or self-efficacy, over others. Nonetheless, this framework enhanced analytical rigor and facilitated the identification of multi-level influences on dietary behavior and diabetes risk.

Despite these limitations, the study provides rich, contextually grounded insights into how traditional food practices intersect with migration, culture, and health perceptions among Cameroonian immigrants. The findings contribute to a growing body of African migrant health.

### Recommendations

Cultural beliefs profoundly shape health perceptions and approaches to disease prevention among immigrant populations (25, 26). Healthcare providers should adopt culturally sensitive care informed by Kleinman’s framework (32, 36–39), prioritizing an understanding of patients’ lived experiences, health perspectives, and cultural priorities. This approach helps minimize miscommunication, build trust, and improve adherence to preventive behaviors, ultimately enhancing health outcomes. Clinical guidance should support nutrient-rich traditional foods, carefully balancing cultural identity with evidence-based dietary recommendations, while avoiding the imposition of foreign dietary norms that may conflict with culturally significant practices.

Community engagement and education initiatives can act as cues to action within the HBM, enhancing perceived susceptibility, perceived benefits, and self-efficacy for T2D prevention. Multi-level strategies guided by the SEM should address behaviors across ecological layers: supporting individual knowledge and skills, strengthening interpersonal networks, shaping community norms, and addressing structural determinants such as food availability, affordability, and access to healthcare services.

Practical interventions may include:

Culturally tailored nutrition workshops demonstrating healthier preparation techniques for traditional Cameroonian foods without compromising taste or cultural meaning.
Educational resources detailing carbohydrate content, nutritional value, and portion control strategies for commonly consumed traditional dishes.
Family- and community-centered programs that foster collective empowerment, strengthen social support, and leverage observational learning to reinforce protective behaviors across multiple generations.
Partnerships with community organizations, faith-based groups, and social networks to disseminate information, encourage peer modeling, and provide ongoing motivation and reinforcement.

Integrating HBM, SEM, risk/protective factors, and Social Cognitive Theory (SCT) ensures that interventions are theoretically grounded, culturally relevant, and practically feasible. Multi-level approaches promote sustainable health behaviors, enhance self-efficacy, respect cultural practices, and reduce T2D risk. Moreover, these strategies may serve as a model for interventions targeting other African migrant populations experiencing similar challenges related to dietary transitions, lifestyle adaptations, and chronic disease prevention.

## Data Availability

The raw data supporting the conclusions of this article will be made available by the authors, without undue reservation.
